# Use of cilomilast-loaded phosphatiosomes to suppress neutrophilic inflammation for attenuating acute lung injury: the effect of nanovesicular surface charge

**DOI:** 10.1186/s12951-018-0364-z

**Published:** 2018-03-30

**Authors:** Fu-Chao Liu, Huang-Ping Yu, Cheng-Yu Lin, Ahmed O. Elzoghby, Tsong-Long Hwang, Jia-You Fang

**Affiliations:** 1 Department of Anesthesiology, Chang Gung Memorial Hospital, Kweishan, Taoyuan, Taiwan; 2 School of Medicine, College of Medicine, Chang Gung University, Kweishan, Taoyuan, Taiwan; 3 Pharmaceutics Laboratory, Graduate Institute of Natural Products, Chang Gung University, 259 Wen-Hwa 1st Road, Kweishan, Taoyuan, 333 Taiwan; 4 Cancer Nanotechnology Research Laboratory (CNRL), Faculty of Pharmacy, Alexandria University, Alexandria, Egypt; 5 Department of Industrial Pharmacy, Faculty of Pharmacy, Alexandria University, Alexandria, Egypt; 6 Research Center for Industry of Human Ecology and Research Center for Chinese Herbal Medicine, Chang Gung University of Science and Technology, Kweishan, Taoyuan, Taiwan; 7 Cell Pharmacology Laboratory, Graduate Institute of Natural Products, Chang Gung University, 259 Wen-Hwa 1st Road, Kweishan, Taoyuan, 333 Taiwan; 8 Chinese Herbal Medicine Research Team, Healthy Aging Research Center, Chang Gung University, Kweishan, Taoyuan, Taiwan

**Keywords:** Cilomilast, Phosphatiosomes, Nanovesicle, Acute lung injury, Surface charge

## Abstract

**Background:**

Cilomilast is a phosphodiesterase 4 (PDE4) inhibitor for treating inflammatory lung diseases. This agent has a narrow therapeutic index with significant adverse effects on the nervous system. This study was conducted to entrap cilomilast into PEGylated phosphatidylcholine-rich niosomes (phosphatiosomes) to improve pulmonary delivery via the strong affinity to pulmonary surfactant film. Neutrophils were used as a cell model to test the anti-inflammatory activity of phosphatiosomes. In an in vivo approach, mice were given lipopolysaccharide to produce acute lung injury. The surface charge in phosphatiosomes that influenced the anti-inflammatory potency is discussed in this study.

**Results:**

The average diameter of the phosphatiosomes was about 100 nm. The zeta potential of anionic and cationic nanovesicles was − 35 and 32 mV, respectively. Cilomilast in both its free and nanocapsulated forms inhibited superoxide anion production but not elastase release in activated neutrophils. Cationic phosphatiosomes mitigated calcium mobilization far more effectively than the free drug. In vivo biodistribution evaluated by organ imaging demonstrated a 2-fold ameliorated lung uptake after dye encapsulation into the phosphatiosomes. The lung/brain distribution ratio increased from 3 to 11 after nanocarrier loading. The intravenous nanocarriers deactivated the neutrophils in ALI, resulting in the elimination of hemorrhage and alveolar wall damage. Only cationic phosphatiosomes could significantly suppress IL-1β and TNF-α in the inflamed lung tissue.

**Conclusions:**

These results suggest that phosphatiosomes should further be investigated as a potential nanocarrier for the treatment of pulmonary inflammation.

## Background

Neutrophils act as the predominant phagocytic cells for the first-line of defense. Although the activation of neutrophils enhances immunity to retard xenobiotic invasion, the overwhelming stimulation contributes to inflammatory disorders and adaptive immune responses [1]. The lung, liver, and spleen are the main reservoirs of neutrophils for rapid deployment to the inflammatory sites. Neutrophil infiltration is observed in acute lung injury (ALI) and in its most severe type, acute respiratory distress syndrome [2]. Neutrophil migration in the lung would release proinflammatory cytokines, disrupting the alveolar epithelium and increasing microvascular permeability [3]. This results in the high mortality rate of about 40%. The causes of ALI include infection, shock, sepsis, ischemia, vasculitis, and drug effect [4]. The neutrophils can be a valid target for ALI treatment. Although steroids have a positive effect on ALI, the drug resistance and side effects that accompany prolonged steroid application necessitate the development of more-specific drugs.

Phosphodiesterase 4 (PDE4) is a cAMP-metabolizing enzyme that plays a key role in the regulation of the inflammatory process. PDE4 inhibitors are investigated as anti-inflammatory agents due to the repressed activity on neutrophil overactivation [5]. Cilomilast is a second-generation PDE4 inhibitor for the treatment of chronic obstructive pulmonary disease (COPD). It is also effective in the therapy of ALI and bronchiolitis obliterans syndrome [6]. Although the anti-inflammatory activity of cilomilast has been well documented, the adverse effects and low therapeutic index remain unsolved problems of clinical application [7]. The distribution of cilomilast to non-target tissues and cells resulted in the failure of phase III clinical trials [8]. Cilomilast causes the central nervous system (CNS) side effects of nausea, vomiting, and headache. Ameliorating cilomilast targeting to the lungs to treat pulmonary inflammation is a potential endeavor.

The nanomedical approach provides the benefit of prolonging the target organ’s retention via active or passive targeting. Active targeting such as antibody and peptide conjugation on the nanoparticulate surface is a powerful strategy for delivering the drugs to the target nidus. However, toxicity concerns and the associated high cost have led to the limitation of extensive application [9]. There are 51 FDA-approved nanomedicines commercially available. To date, only one is an active targeting nanosystem (Ontak^®^) [10]. Passive targeting by modulation of nanoparticulate materials and structures for increased affinity to target tissues or cells is a choice for efficient drug delivery. We had previously developed phosphatiosomes for passive targeting to the lungs mediated by the enhanced pulmonary surfactant affinity [11]. Phosphatiosomes are a niosomal nanocarrier rich in phosphatidylcholine (PC) and distearylphosphatidylethanolamine–polyethylene glycol (DSPE–PEG). Another advantage of phosphatiosomes is the rich PC in the nanovesicles for supplying insufficient pulmonary surfactants in ALI. The aim of this study was to suppress neutrophilic inflammation to attenuate ALI using cilomilast-loaded phosphatiosomes. To adapt the observation to the clinical condition, we employed isolated human neutrophils as the model cells to evaluate the effect of phosphatiosomes on inflammation inhibition. We explored the effect of the nanosystems on a lipopolysaccharide (LPS)-elicited mouse model of ALI, which is a neutrophil-dominant inflammation model. The targeting ability of phosphatiosomes to the lung was monitored by an in vivo imaging system. The chemical surface modulation of the nanoparticles allows significant change to neutrophil interaction and airway delivery [12]. The surface charge of the phosphatiosomes was the variable used in the present study to compare the impact on biodistribution and therapeutic efficiency.

## Methods

### Preparation of phosphatiosomes

A thin-film preparation was used to fabricate the phosphatiosomes. The detailed protocol was described previously [11]. The ingredients used for the anionic phosphatiosomes were Span 60 (0.3%, w/v), cholesterol (0.3%), soybean PC (Phospholipon^®^ 80H, 0.2%), and DSPE–PEG (5000 Da, 0.4%) in double-distilled water. Soyaethyl morphonium ethosulfate (SME, 1%) was added to the nanosystems to produce cationic phosphatiosomes. Cilomilast (0.125%) as the active ingredient was incorporated into the nanovesicles.

### Vesicle size and zeta potential

The average diameter and surface charge of the phosphatiosomes were measured using a laser-scattering method (Nano ZS90, Malvern). The final phosphatiosome product was diluted 100-fold with double-distilled water prior to determination.

### Molecular environment

The molecular environment (polarity) of the nanovesicles was examined by fluorescence spectrophotometry (F2500, Hitachi) based on the solvatochromism of Nile red. Phosphatiosomes were loaded with Nile red (1 ppm). The emission spectra of the dye-loaded phosphatiosomes were scanned from 550 to 700 nm. The excitation wavelength was 546 nm.

### Cilomilast release from phosphatiosomes

The cilomilast release measurement was conducted using Franz diffusion cells. A cellulose membrane (Cellu-Sep^®^ T2) with a molecular weight cutoff of 6000 – 8000 Da was used as the release dialysis wall. The receptor compartment (5.5 ml) contained 30% ethanol in pH 7.4 citrate–phosphate buffer. The donor (0.5 ml) was loaded with control solution (DMSO) or phosphatiosomes containing cilomilast. The available release area was 0.785 cm^2^. The receptor temperature was set at 37 °C. The 300-μl aliquots of the receptor medium were withdrawn at determined intervals, and then immediately added to an equal volume of fresh medium. The samples taken from the receptor were quantified by ultraviolet spectrophotometry (U3010, Hitachi).

### Isolation of human neutrophils

The protocol was approved by the Institutional Review Board at Chang Gung Memorial Hospital, and written informed consent was obtained from each volunteer. Whole blood was withdrawn from healthy volunteers from ages 20 to 30 years old. Human neutrophils were isolated using a typical method of dextran sedimentation prior to centrifugation in a Ficoll-Hypaque gradient and hypotonic lysis of erythrocytes [13]. The granulocyte layer was harvested and resuspended in Ca^2+^-free HBSS at pH 7.4, which was maintained at 4 °C till use.

### Measurement of superoxide anion (O_2_^·−^) production

The reduction of ferricytochrome *c* was employed to assess O_2_^·−^ release from the neutrophils [14]. The neutrophils (6 × 10^5^ cells/ml) were incubated with 0.5 mg/ml ferricytochrome *c* and 1 mM CaCl_2_ at 37 °C, and were then treated with free cilomilast control or cilomilast-loaded phosphatiosomes for 10 min. Neutrophils were stimulated by adding formyl-methionyl-leucyl phenylalanine (fMLF, 0.1 μM) with cytochalasin B (1 μg/ml). The reduction of ferricytochrome *c* was monitored by the absorbance at 550 nm using an ultraviolet spectrophotometer (U3010).

### Measurement of elastase release

The neutrophils (6 × 10^5^ cells/ml) were equilibrated with the elastase substrate MeO-Suc-Ala-Ala-Pro-Val-*p*-nitroanilide (100 μM) at 37 °C for 2 min. Subsequently, the cilomilast solution or phosphatiosomes were added into the suspension for 10 min. The neutrophils were activated by fMLF and cytochalasin B for a further 10 min. Elastase was measured by detecting the change of absorbance at 405 nm.

### Measurement of lactate dehydrogenase (LDH)

LDH was an indicator of cellular membrane leakage and cytotoxicity. A commercially available kit (CytoTox 96^®^, Promega) was used to measure the LDH content. The cells were treated with the control solution or phosphatiosomes for 17 min. The resulting suspension was centrifuged at 200×*g* and 4 °C for 8 min. The LDH assay reagent was added to the supernatant. The LDH release was measured by ELISA at 492 nm. The enzyme liberated following incubation with 0.1% Triton X-100 was regarded as total LDH release (100%).

### Measurement of intracellular calcium ([Ca^2+^]_i_)

The cells (3 × 10^6^ cells/ml) were treated with Fura-3/AM (2 μM) at 37 °C for 30 min, followed by centrifugation and resuspension in HBSS containing CaCl_2_ (1 mM). The neutrophils were treated with cilomilast-loaded formulations for 5 min. The [Ca^2+^]_i_ in response to fMLF was detected under continuous stirring using a fluorescence spectrophotometer with the excitation and emission wavelength of 488 and 520 nm, respectively.

### Nanovesicle uptake by neutrophils

Phosphatiosomes were labeled with 0.1 mg/ml rhodamine 800 as a fluorescence dye to visualize the uptake by human neutrophils. The cells (1.8 × 10^7^ cells/ml) were incubated with phosphatiosomes at 37 °C for 5 min. HBSS was added into the suspension to terminate the reaction at 4 °C. Confocal microscopy (TCS SP2, Leica) was employed to monitor the phagocytosis of the phosphatiosomes in the neutrophils by a two-dimensional fashion.

### Animals

Male C57BL/6 mice (20 – 25 g) purchased from the National Laboratory Animal Center (Taipei, Taiwan) were used in the in vivo biodistribution and pharmacodynamic experiments. The protocol was received and approved by the Institutional Animal Care and Use Committee of Chang Gung University. All animals were housed and handled according to the institutional guidelines.

### Biodistribution

The iFluor^®^ 790 (0.08%, w/v) was used as the near-infrared (NIR) dye for monitoring the biodistribution of intravenous phosphatiosomes. The control solution of iFluor^®^ 790 (DMSO:Tween80:water = 2:1:2) was used for comparison. The mice were anesthetized using Zoletil^®^ 50 (30 mg/kg) and xylazine (6 mg/kg). The mice were intravenously administered via the tail vein with the control solution or phosphatiosomes at a dose of 2 ml/kg. After 2 h, the mice were sacrificed and the organs were excised. The NIR signal in the organs was observed using the Pearl^®^ Impulse Imaging System (Li-Cor). The quantification of NIR intensity was processed by Pearl^®^ Impulse software.

### ALI mouse model induced by LPS

The animals were randomly divided into seven groups with six mice in each: (i) sham-operated mice treated with the control vehicle; (ii) ALI mice treated the with control vehicle; (iii) ALI mice treated with cilomilast in the control vehicle; (iv) ALI mice treated with anionic phosphatiosomes without cilomilast; (v) ALI mice treated with anionic phosphatiosomes with cilomilast; (vi) ALI mice treated with cationic phosphatiosomes without PDE4 inhibitor; and (v) ALI mice treated with cationic phosphatiosomes with the inhibitor. We intravenously administered the vehicle or phosphatiosomes at a cilomilast dose of 2.5 mg/kg. After 30 min, LPS (10 mg/kg) or normal saline (sham control, 50 μl) was injected into the airway via the tracheostomy [15]. At 6 h after LPS administration, the lung was resected for an assay of immunohistochemistry (IHC) and cytokines.

### Histological visualization

The lung specimen was immersed in 10% buffered formaldehyde using ethanol, embedded in paraffin wax, and sliced at a thickness of 3 μm. The specimen was stained by hematoxylin and eosin (H&E) and observed by optical microscopy (IX81, Olympus). IHC was carried out to stain myeloperoxidase (MPO) and lymphocyte antigen 6 complex locus G6D (Ly6G) in the lung tissue. The detailed protocol for IHC staining was described previously [15].

### Measurement of MPO and cytokines in lung tissue

The resected lung tissue was weighed and homogenized in 1-ml lysis buffer (0.5% hexadecyltrimethylammonium bromide in 50 mM phosphate buffer) by MagNA Lyser (Roche). After centrifugation at 12,000×*g* for 20 min, the supernatant was collected for assay. The expression level of MPO and cytokines was determined with enzyme immunoassays according to the manufacturer’s protocols.

### Statistical analysis

The data are presented as mean and standard error of the mean (SEM). The difference in the data of the different treatment groups was assayed using the Kruskal–Wallis test. The post hoc test for checking individual differences was the Dunn’s test. Significance was indicated as * for *p* < 0.05, ** for *p* < 0.01, and *** for *p* < 0.001.

## Results

### Physicochemical properties of phosphatiosomes

The nanocarriers were produced in the absence (anionic phosphatiosomes) and presence of SME (cationic phosphatiosomes). Table 1 summarizes the diameter, polydispersity index (PDI), and surface charge of the nanovesicles. After preparation, the anionic phosphatiosomes exhibited a hydrodynamic diameter of 121 nm. The SME incorporation led to the reduction in size to 100 nm. This could be due to the surfactant property of SME. Both nanosystems revealed stable unimodal size distribution with PDI of ≤ 0.25, demonstrating a narrow distribution. Negatively charged nanocarriers were achieved (− 35 mV) for anionic phosphatiosomes, whereas the SME addition generated positively charged vesicles (32 mV). A zeta potential of > ±30 mV is required for a physically stable nanosystem. Phosphatiosomes fitted this criterion.

**Table 1:** The vesicle size, polydispersity index (PDI) and zeta potential of cilomilast-loaded phosphatiosomes

Formulation	Size (nm)	PDI	Zeta potential
Anionic phosphatiosomes	120.67 ± 0.27	0.20 ± 0.01	− 34.60 ± 0.06
Cationic phosphatiosomes	100.29 ± 0.32	0.25 ± 0.003	32.43 ± 0.72

Each value represents the mean ± SEM (*n *= 3)

**Fig. 1:**
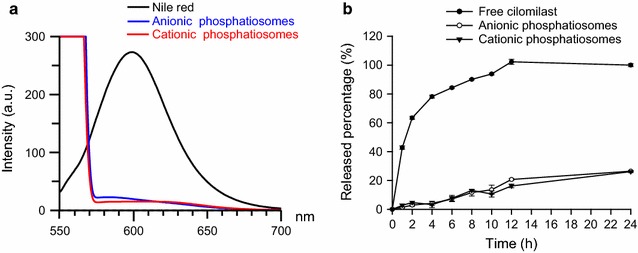
Comparison of physicochemical properties of various phosphatiosomes: **a** the polarity of anionic and cationic squarticles determined by the solvatochromism of Nile red, and **b** in vitro released amount-time curves of cilomilast from control solution and phosphatiosomes. Each value represents the mean ± SEM (*n *= 4)

Nile red acts as a fluorescence marker that can change the emission with environmental polarity after nanoparticulate entrapment. Figure 1a depicts the emission spectra of Nile red in acetone and phosphatiosomes. Nile red in acetone showed a very high fluorescence intensity due to the strong lipophilicity of the organic solvent. Encapsulation of Nile red into the phosphatiosomes resulted in a great reduction of intensity, suggesting a more hydrophilic nature of the nanosystems. The aqueous core inside the surfactant-lipid bilayer membrane of the phosphatiosomes may contribute to the hydrophilic character. The intensity between anionic and cationic nanovesicles was comparable. Figure 1b illustrates the release percentage of cilomilast from the control solution and the phosphatiosomes. Cilomilast displayed an initial burst release from the control. After 10 h, free cilomilast release reached a maximum. The drug molecules were completely released by 24 h. The nanovesicular encapsulation enabled the sustained and controlled release of cilomilast. The release pattern of the phosphatiosomes followed the zero-order kinetics. Only 26% of cilomilast was released from the nanocarriers at 24 h.

### Anti-inflammatory activity of phosphatiosomes in activated neutrophils

**Fig. 2:**
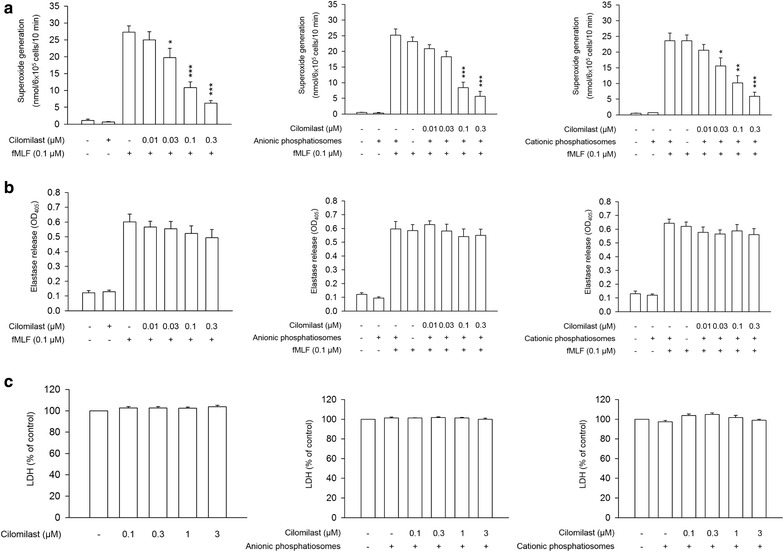
Anti-inflammatory activity of cilomilast in control solution and phosphatiosomes against fMLF-activated neutrophils: **a** superoxide anion production, **b** elastase release, and **c** LDH leakage. Each value represents the mean ± SEM (*n *= 6)

O_2_^·−^ production, elastase release, and LDH leakage are indicators of neutrophilic inflammation. Figure 2 shows the anti-inflammatory effect of cilomilast in free form and nanovesicular form on these indicators. Free cilomilast did not influence basal O_2_^·−^ in the neutrophils without fMLF stimulation (Fig. 2a). The superoxide level increased 42-fold after the fMLF challenge. Treatment of free cilomilast significantly and concentration-dependently decreased the fMLF-induced O_2_^·−^. No O_2_^·−^ burst was detected when the neutrophils were exposed to phosphatiosomes without activation. Incubation of fMLF-stimulated neutrophils in the presence of phosphatiosomes resulted in a dose-dependent inhibition on superoxide anion. Free cilomilast, anionic phosphatiosomes, and cationic phosphatiosomes in a similar trend inhibited O_2_^·−^ production. The IC_50_ value of the free drug, anionic, and cationic nanocarriers was 0.07 ± 0.02, 0.08 ± 0.01, and 0.08 ± 0.02 μM, respectively.

fMLF elevated elastase release from the neutrophils (Fig. 2b). The treatment of free cilomilast and nanocarriers exhibited no marked inhibition of elastase. This suggested that this PDE4 inhibitor had no function on the degranulation of activated neutrophils. A cytotoxicity experiment of LDH leakage was investigated to examine whether the inhibitory activity of cilomilast was mediated by the cytotoxic effect. As shown in Fig. 2c, neither the free drug nor drug-loaded phosphatiosomes influenced the cell viability. Even treating neutrophils with a high concentration of cilomilast (3 μM) failed to display any suppression of viability. The result suggested that the anti-inflammatory activity on the respiratory burst was not the consequence of cytotoxicity.

**Fig. 3:**
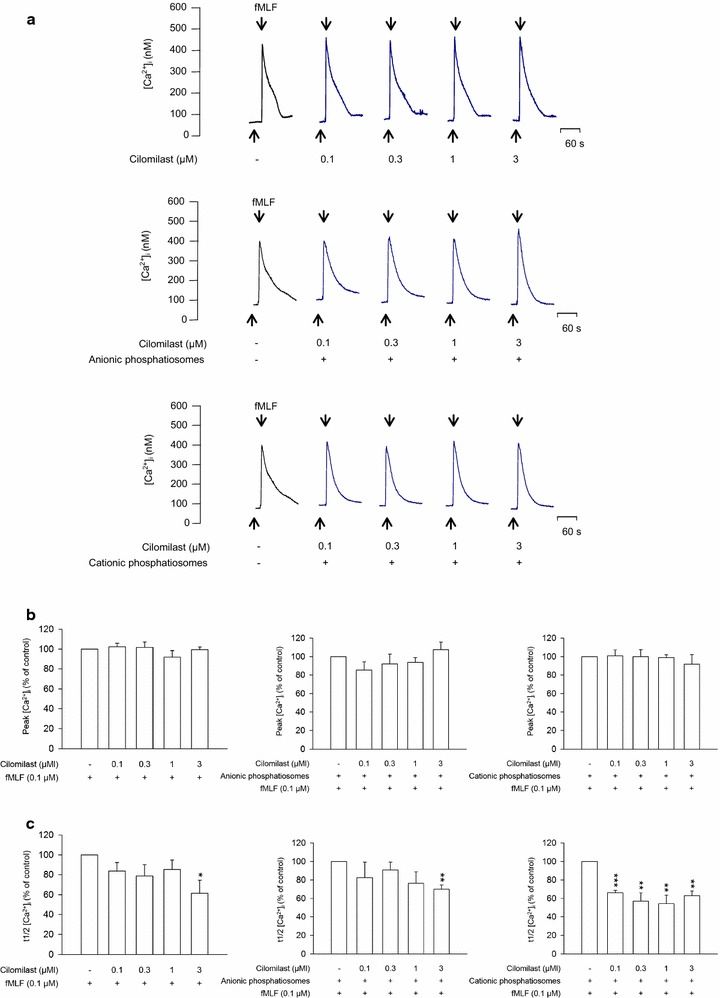
The calcium mobilization of fMLF-activated neutrophils after treatment of cilomilast in control solution and phosphatiosomes: **a** the [Ca^2+^]_i_-time curves, **b** the peak [Ca^2+^]_i_ concentration, and **c** the time required for peak [Ca^2+^]_i_ to return to half (t_1/2_). Each value represents the mean ± SEM (*n *= 6)

Ca^2+^ is a second messenger in neutrophil stimulation. The [Ca^2+^]_i_ is crucial in the regulation of O_2_^·−^ and elastase in activated neutrophils. fMLF evokes a rapid and transient increase in intracellular calcium as shown in Fig. 3a. Free and anionic nanovesicular cilomilast failed to inhibit [Ca^2+^]_i_ mobilization and peak concentration. On the other hand, cilomilast-loaded cationic nanocarriers could accelerate the resequestration of calcium, although the peak concentration was not altered. As depicted in Fig. 3b, there was no significant difference in peak [Ca^2+^]_i_ between the activated neutrophils treated with and without cilomilast. The time required for peak [Ca^2+^]_i_ to return to half (t_1/2_) was calculated as shown in Fig. 3c. The data showed that cationic phosphatiosomes had shorter t_1/2_ compared to the free control at all concentrations tested. This indicated a greater anti-inflammatory effect of the cationic nanocarriers than the free molecules. We found no reduction of t_1/2_ for the drug in the free and anionic nanovesicular forms except at the highest concentration (3 μM), which exhibited a significant decrease.

**Fig. 4:**
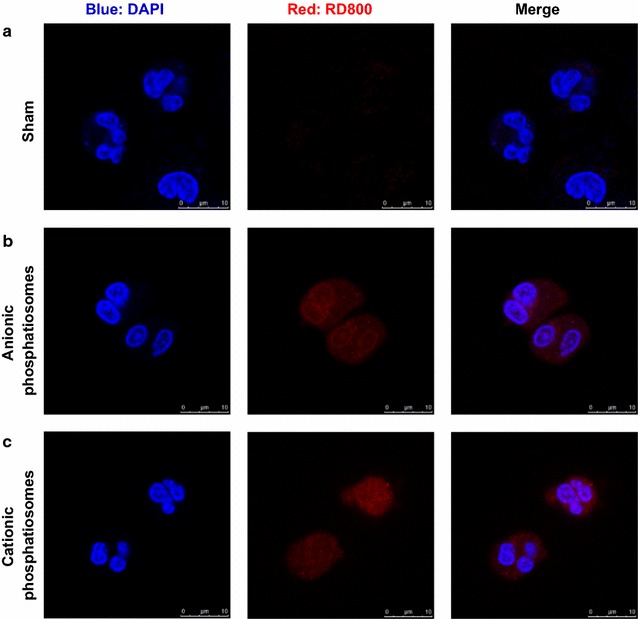
The cellular uptake of rhodamine 800-loaded phosphatiosomes into fMLF-activated neutrophils examined by confocal laser scanning microscopy: **a** sham control, **b** anionic phosphatiosomes, and **c** cationic phosphatiosomes. The blue signal is the nucleus stained by DAPI. The red signal is rhodamine 800

We evaluated the capability of human neutrophils to internalize phosphatiosomes using confocal microscopy. The confocal imaging in Fig. 4a shows the untreated neutrophils. The nucleuses were stained with DAPI to display blue color. The autofluorescence was negligible in the neutrophil cytosol. The neutrophils treated with anionic phosphatiosomes exerted a homogeneous red fluorescence in the nucleuses and cytosol (Fig. 4b), demonstrating a facile uptake of anionic nanovesicles into the neutrophils. The confocal images of the neutrophils incubated with cationic nanocarriers revealed comparable fluorescence with anionic formulations in the cytoplasm.

### Biodistribution of phosphatiosomes

**Fig. 5:**
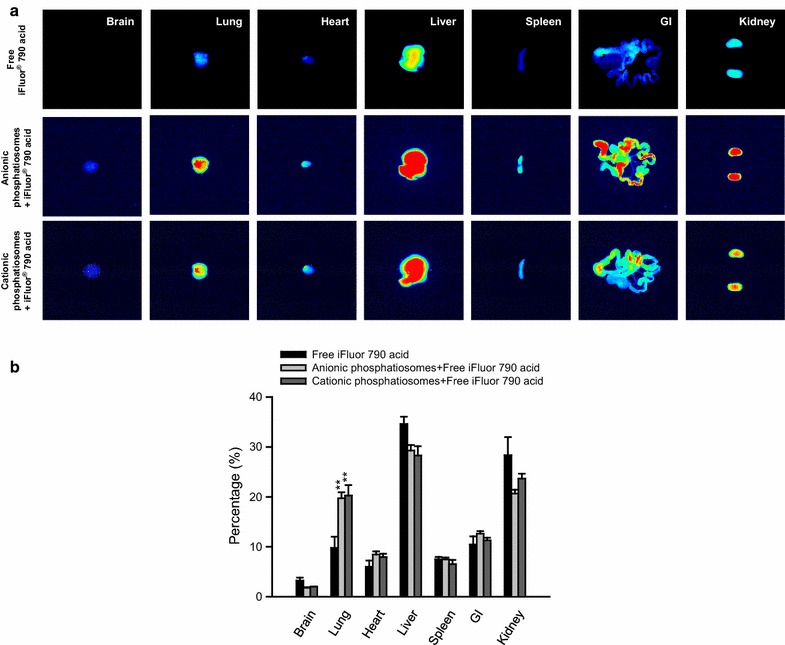
The bioimaging of seven peripheral organs from the mice at 2 h following an intravenous injection of iFluor^®^ 790 loaded in a control solution and in phosphatiosomes: **a** the representative organ images and **b** the percentage of near-infrared signal in different organs. The scale of bioimaging was calibrated by the intensity of the formulations for impartial comparison. Each value represents the mean ± SEM (*n *= 5)

We compared the bulk organ distribution of anionic and cationic phosphatiosomes after intravenous injection in mice. Nanovesicles were fabricated with the incorporation of NIR dye to enable visualization throughout the organs. The rats receiving normal saline injection exhibited a negligible auto-fluorescence in the organs (data not shown). The NIR signal of the organs from free or nanovesicular dye-treated groups was depicted in Fig. 5a after subtracting the auto-fluorescence. The NIR intensity of the organs was calibrated by the intensity of dye-labeled formulations resulting from different NIR signals in different formulations. As shown in Fig. 5a, free dye in the control solution was mainly stored in the liver, followed by the kidney and lung. Both phosphatiosomes were observed throughout all the organs, which exhibited higher NIR intensity than the free control. This suggested a preferred transfer of intravenous nanovesicles from the circulation to the peripheral organs. The anionic phosphatiosomes showed greater accumulation in the lung and kidney as compared to the cationic ones. The NIR intensity was quantified to calculate the distribution percentage in various organs as shown in Fig. 5b. The greatest uptake for all formulations was found in the liver. A significantly greater percentage of phosphatiosomes (about 20%) was measured in the lung compared with the free control. A 2-fold increase was detected while comparing the nanovesicles and the free dye. Inclusion of the dye in the nanocarriers reduced the accumulation percentage in the brain and kidney; however, this reduction did not achieve a statistical significance.

### LPS-induced ALI treated by phosphatiosomes

**Fig. 6:**
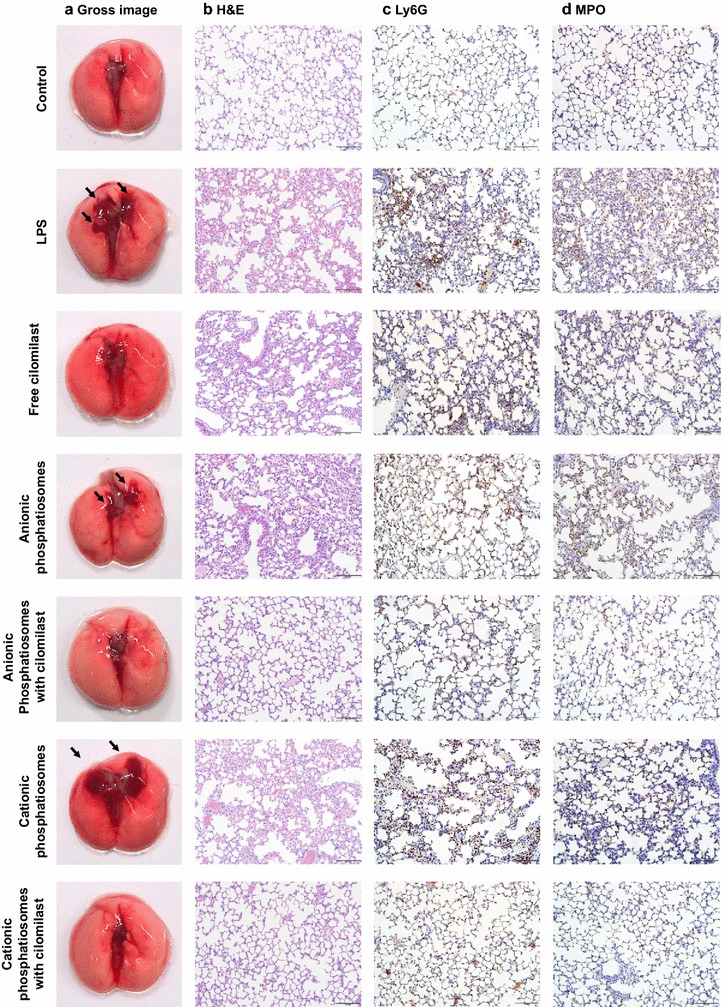
The lung histology of LPS-challenged mice treated by cilomilast in control solution and phosphatiosomes: **a** gross appearance, **b** H&E staining, **c** Ly6G antibody staining, and **d** MPO antibody staining

The anti-inflammatory effect of cilomilast and its nanovesicular form was examined in a mouse model of ALI. Intratracheal LPS to mice is a well-accepted model for ALI with neutrophilic inflammation. At 6 h after the LPS challenge in the mice, the animals were sacrificed to evaluate the lung histology and cytokines. As presented in Fig. 6a, the untreated lung showed no damage in gross appearance. In contrast, LPS administration resulted in a large area of edema and hemorrhage in the lung (arrows in Fig. 6a). The injection of cilomilast-loaded solution and phosphatiosomes could alleviate the hemorrhage. The plain phosphatiosomes without the drug were also examined in this experiment. We found no hemorrhage abatement in the lung treated with the plain phosphatiosomes. The H&E staining of the untreated control lung revealed a normal structure (Fig. 6b). LPS caused inflammatory cell infiltration, alveolar hemorrhage, and alveolar wall thickening. These pathological features could be greatly improved by cilomilast-loaded phosphatiosomes. The improvement was less pronounced after free cilomilast administration. The inflammatory symptoms induced by LPS were not modified by phosphatiosomes lacking cilomilast.

**Fig. 7:**
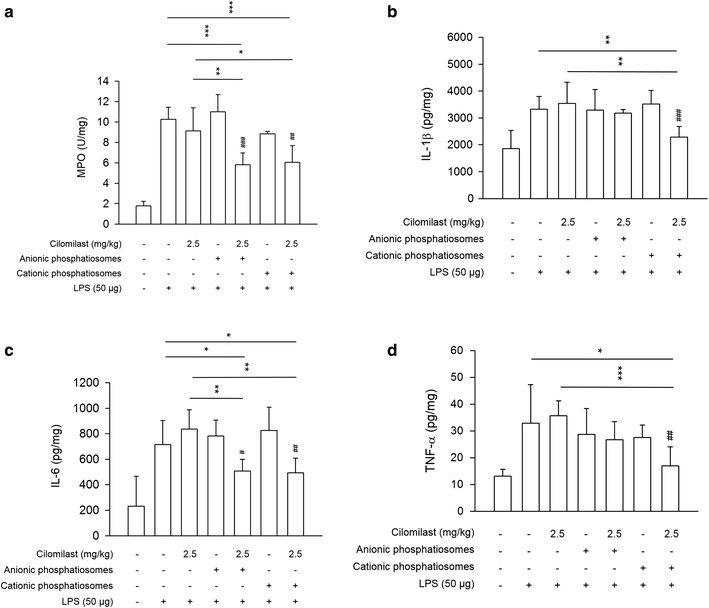
The biomarkers in lung of LPS-challenged mice treated by cilomilast in control solution and phosphatiosomes: **a** MPO, **b** IL-1β, **c** IL-6, and **d** TNF-α. Each value represents the mean ± SEM (*n *= 6)

We further assessed the ALI-induced inflammation by staining Ly6G antibody for neutrophil labeling as shown in Fig. 6c. The images demonstrated a greater number of neutrophils in the parenchyma of the LPS-treated group compared to the untreated control. The treatment with cilomilast-loaded formulations reduced the neutrophilia, with the nanocarriers displaying the better attenuation. Again, the plain phosphatiosomes showed no inhibition of neutrophil infiltration. During the process of neutrophil recruitment, MPO was rapidly released upon stimulation. Figure 6d indicates a much higher degree of MPO in lung tissue compared with the sham control. The same as the result of Ly6G, treatment with drug-containing nanovesicles clearly decreased the MPO level induced by LPS. The decrease of MPO activity was limited for the free cilomilast group. We also quantified the MPO level to explore the effect of phosphatiosomes on inflammation. As shown in Fig. 7a, the MPO content in the LPS-treated lung had a significant 6-fold increase compared with the sham control. Injection of the free drug did not inhibit the expression. Following administration of cilomilast phosphatiosomes, MPO was significantly abolished. This trend was similar to that in the histological result.

The proinflammatory cytokines IL-1β, IL-6, and TNF-α are the promising biomarkers for assessing the morbidity of ALI. Our data showed a much higher level of cytokines after the LPS challenge than the sham control (Fig. 7b–d). Cilomilast in the control solution and anionic phosphatiosomes did not reduce IL-1β and TNF-α in the lung, but they were decreased by cationic phosphatiosome treatment. This result indicated that cationic nanoformulations exhibited a stronger action on inflammation abatement than anionic phosphatiosomes. IL-6 decreased in response to administration of both phosphatiosomes at a similar level. The inhibitory effect on MPO and cytokine production was absent for the plain phosphatiosomes without the drug.

## Discussion

Neutrophil influx mediates ALI through certain mechanisms such as superoxide generation, elastase release, and production of cytokines. The overactivation of neutrophils contributes to pulmonary edema and epithelial injury. The development of a therapeutic approach is still a challenge for ALI. The pulmonary surfactants form a thin lipid-protein film that lines the lung surface. They can be used as an efficient medium for delivering drugs deep into the lung [16]. We developed anti-ALI phosphatiosomes to interact with pulmonary surfactants to passively target the lung. Nanovesicular cilomilast maintained the anti-inflammatory activity against the activated neutrophils to a greater degree than the free drug. The biodistribution profile clearly demonstrated a higher accumulation of intravenous phosphatiosomes in the lung compared to the free dye, indicating that the nanocarriers could enhance pulmonary targeting. This study showed that treatment of cilomilast-loaded phosphatiosomes mitigated the inflammatory response and pathological signs in ALI.

Neutrophil migration into the lung is insufficient for generating ALI. Another requirement is the activation of neutrophils. The activated neutrophils damage the lung through the production of certain substances, including reactive oxygen species (ROS), serine proteases, and cytokines [17]. A respiratory burst of neutrophils is an oxygen-dependent process leading to the formation of ROS. The level of oxidative stress correlates well with ALI severity [2]. Our result suggested a marked mitigation of O_2_^·−^ in fMLF-activated neutrophils by cilomilast. The blockade of PDE4 to attenuate LPS-induced ROS was approved in the previous study [18]. The capability of cilomilast to abolish O_2_^·−^ could be reserved after nanovesicular entrapment. ROS generation is an important step in neutrophil phagocytosis [19]. The confocal images illustrated a facile internalization of phosphatiosomes into the nucleuses and cytosol of the neutrophils, with cationic nanosystems showing a higher uptake. This could be due to the affinity of cationic nanovesicles with the negatively charged neutrophil membrane.

fMLF excites neutrophils by binding to the G protein-coupled receptor. This stimulation induces the increase of [Ca^2+^]_i_, initiating the inflammatory response of respiratory burst and degranulation [20]. Our study showed that cilomilast itself could not reduce fMLF-induced [Ca^2+^]_i_. Cilomilast also had no function in suppressing elastase release. This demonstrates a crucial role of [Ca^2+^]_i_ in regulating the degranulation pathway. Cilomilast in the cationic nanovesicular form could accelerate the process of [Ca^2+^]_i_ resequestration. The facile uptake of cationic phosphatiosomes into the neutrophils delivered a sufficient amount of cilomilast inside the cells to trigger the PDE4 blockade. Phosphatiosomes did not change the O_2_^·−^ in the unstimulated cells. The LDH result also showed a negligible cytotoxicity of the nanovesicles on the neutrophils. These results suggest that phosphatiosomes have few or no toxic effects disruptive to neutrophils’ homeostasis.

LPS acts on Toll-like receptor 4 to promote cytokines in the lung, resulting in neutrophil infiltration, hemorrhage, and alveolar disruption [21]. The cytokines are early biomarkers that respond in ALI. We found that cilomilast-loaded phosphatiosomes suppressed pulmonary hemorrhage, neutrophil infiltration, and parenchymal damage in ALI. Phosphatiosomes also decreased MPO and cytokines in the inflamed lung, with the cationic nanocarriers showing more effective activity. In contrast to the result of phosphatiosomes, free cilomilast had less inhibition on ALI. The previous study [22] also demonstrated the lack of inhibition by cilomilast on LPS-induced cytokines. Due to the higher binding to plasma proteins (99.4%), cilomilast distributes minimally into the peripheral organs with a small volume of distribution [23]. This could lead to limited delivery to the target organs, thereby lessening the degree of therapeutic activity exhibited. The inclusion of cilomilast inside the nanovesicles may lessen this interaction, thus increasing the distribution to the peripheral organs. The biodistribution profile revealed a significant phosphatiosome accumulation in the organs. Phosphatiosomes showed a facile uptake to the liver. Most nanoparticles 10 – 300 nm in size are nonspecifically cleared by the reticuloendothelial system such as the liver and spleen [24].

The biodistribution result also demonstrated a large number of nanovesicles in the lung, the target organ for treating ALI. Upon deep pulmonary deposition, the pulmonary surfactant layer is the important interface that the nanoparticles encounter [25]. The introduction of abundant PC into the phosphatiosomes was key to achieving pulmonary targeting. The affinity of phosphatiosomes to pulmonary surfactants was affirmed in our previous work [11]. The PC adsorbs to the surface-active aggregates at the air–liquid interface of alveoli [26]. It is recognized that PC strongly interacts with dipalmitoylphosphatidylcholine (DPPC), the main component of pulmonary surfactants, via hydrogen and hydrophilic bonding [27]. The neutrophil migration and the subsequent ROS release disorganize the tight junctions of the pulmonary endothelium to increase the permeability of the alveolar capillary barrier in the case of ALI [2]. The effortless delivery of nanovesicles into the lung could be due to the enhanced permeability and retention (EPR) effect prompted by the inflammation present in ALI. It is inferred that the nanovesicles were trapped by the damaged pulmonary capillary network and further transported through the pulmonary surfactant film into the alveoli where PDE4 inhibitor could take effect on the activated neutrophils. The previous study [28] suggested that hydrophilic nanoparticles tended to translocate rapidly across the pulmonary surfactant layer. The result of molecular polarity confirmed a high hydrophilicity of phosphatiosomes. Previous investigations [29, 30] also showed a strong affinity of cationic amphiphiles to DPPC. This result was identical to the finding that cationic phosphatiosomes resulted in greater improvement of ALI than did anionic ones. Another possibility behind the superior effect of cationic phosphatiosomes was the smaller size compared to the anionic nanosystems, since the smaller nanoparticles possess a greater affinity to the surfactant film [31].

Cilomilast has a strong CNS action that generates side effects such as emesis and headache. The intravenous phosphatiosomes enhanced pulmonary distribution but reduced the uptake in the brain compared to the free dye. The lung/brain distribution ratio was 3.0, 11.0, and 10.1 for the free control, anionic, and cationic phosphatiosomes, respectively. The nanomedical approach is helpful for increasing the effectiveness of pulmonary therapy and decreasing the toxicity of the nervous system. The therapeutic index of cilomilast was thus increased. At the cellular level, a nanoparticulate size of nearly 100 nm is suitable for facilitating the uptake by the mammalian cells in the ALI condition [32]. The superior neutrophil internalization and sustained cilomilast release could efficiently improve the inflammatory and pathological signs of ALI. The enhanced cellular uptake of phosphatiosomes might trigger the [Ca^2+^]_i_ sequestration, thus dampening the inflammation. Cilomilast release from the phosphatiosomes was extremely low compared to that with the free control. Phosphatiosomes were beneficial to protecting the drug by encapsulation, minimizing the metabolism and increasing pulmonary delivery. As a large number of phosphatiosomes entered the activated neutrophils in the lung, the nanovesicles might decompose by the phagolysosomes, enabling the drug release in the cytosol to trigger pharmacological activity. Phagolysosomes are an important cytoplasmic body for disintegrating the xenobiotic colloids in neutrophils [33].

Both ALI and COPD cause neutrophil-dominant inflammation in the lung [34]. Phosphatiosomes may also be used to treat COPD to extend the application. Although the nanocarriers could diminish the accumulation in the brain for possible minimization of side effects, further study is needed to confirm this inference. Although cationic phosphatiosomes seemed to play a role in superior inflammation inhibition compared to anionic ones based on the ability to significantly repress IL-1β and TNF-α, the influence of the surface charge was not large. Further modification of the nanoformulations is necessary. How to decrease the RES uptake of phosphatiosomes is another concern, which also can be resolved by formulation design.

## Conclusions

We developed the anionic and cationic phosphatiosomes entrapped with PDE4 inhibitor for pulmonary delivery to treat ALI. The inhibitory effect of cilomilast on superoxide anion produced by neutrophils could be maintained after encapsulation into the phosphatiosomes. The biodistribution study showed that phosphatiosomes preferentially accumulated in the lung with an increased lung/brain ratio. Intravenously administered phosphatiosomes would offer enhanced lung targeting and possibly minimize the adverse effects for treating lung inflammation. Phosphatiosomes alleviated hemorrhage, alveolar wall damage, and neutrophil trafficking caused by ALI. The ALI-related biomarkers of MPO and cytokines were also reduced by the nanocarriers, whereas the free cilomilast showed no effect on these indicators. The cationic nanosystems showed better amelioration on ALI than anionic ones because the cationic but not anionic phosphatiosomes could significantly mitigate IL-1β and TNF-α in the lung tissue. The facile distribution to the peripheral organs, the affinity of the nanocarriers to pulmonary surfactant film, and the protection of the drug by the nanocarriers contributed to the superior therapy of ALI by phosphatiosomes. The cilomilast-loaded nanovesicles also exhibited significant cell internalization and [Ca^2+^]_i_ resequestration for inhibiting neutrophilic activation. The experimental results of this study suggest that phosphatiosomes are an attractive PDE4 inhibitor carrier for treating pulmonary inflammation.
